# PD-1/PD-L Axis in Neuroinflammation: New Insights

**DOI:** 10.3389/fneur.2022.877936

**Published:** 2022-06-09

**Authors:** Susanna Manenti, Mario Orrico, Stefano Masciocchi, Alessandra Mandelli, Annamaria Finardi, Roberto Furlan

**Affiliations:** ^1^Clinical Neuroimmunology Unit, Division of Neuroscience, Institute of Experimental Neurology, IRCCS Ospedale San Raffaele, Milan, Italy; ^2^Neuroimmunology Laboratory and Research Unit, IRCCS Mondino Foundation, Pavia, Italy

**Keywords:** PD-1, PD-L1, PD-L2, neuroinflammation, multiple sclerosis, Alzheimer's disease

## Abstract

The approval of immune checkpoint inhibitors (ICIs) by the Food and Drug Administration (FDA) led to an improvement in the treatment of several types of cancer. The main targets of these drugs are cytotoxic T-lymphocyte antigen 4 (CTLA-4) and programmed cell death protein-1/programmed death-ligand 1 pathway (PD-1/PD-L1), which are important inhibitory molecules for the immune system. Besides being generally safer than common chemotherapy, the use of ICIs has been associated with several immune-related adverse effects (irAEs). Although rare, neurological adverse effects are reported within the irAEs in clinical trials, particularly in patients treated with anti-PD-1 antibodies or a combination of both anti-CTLA-4 and PD-1 drugs. The observations obtained from clinical trials suggest that the PD-1 axis may play a remarkable role in the regulation of neuroinflammation. Moreover, numerous studies in preclinical models have demonstrated the involvement of PD-1 in several neurological disorders. However, a comprehensive understanding of these cellular mechanisms remains elusive. Our review aims to summarize the most recent evidence concerning the regulation of neuroinflammation through PD-1/PD-L signaling, focusing on cell populations that are involved in this pathway.

## Introduction to Neuroinflammation

Immune checkpoints, such as programmed cell death protein-1 (PD-1) and its ligands, are regulatory molecules that are fundamental to suppress the immune response and promote self-tolerance. PD-1 has two known ligands: PD-L1 (also called B7 homolog 1, B7-H1) and PD-L2 (or B7-DC). Both ligands have been characterized as powerful inhibitors in the context of tumor evasion from the immune system. Since 2014, six different inhibitors of PD-1 and PD-L1 were approved for cancer immunotherapy by the US Food and Drug Administration (FDA) and the European Medicines Agency (EMA) (1), revolutionizing the treatment of certain cancers. However, in addition to their desired effects, immune-checkpoints inhibitors (ICIs) modify the balance of immune responses and induce specific off-target toxicities called immune-related adverse events (irAEs) (2). Several neurological immune-related adverse events were described during post-marketing surveillance with an estimated incidence of about 3–4% (3, 4). The reported neurological irAEs include encephalitis, aseptic meningitis, peripheral neuropathy, myasthenia gravis, and myositis. These clinical observations, combined with growing evidence about the role of PD-1 in neuroinflammatory disorders, suggest that the PD-1 axis may play a critical role not only in peripheral immune imbalance but also in the regulation of neuroinflammation, as highlighted in a previous work by Zhao et al. (5, 6). A comprehensive view of the cell-to-cell interactions and the molecular mechanisms underlying PD-l functions in neuroinflammation is, however, still missing. This review aims to summarize the most recent evidence concerning the regulation of human and murine neuroinflammatory disorders by PD-1 signaling, focusing on cell populations that are involved in this pathway.

## PD-1/PD-L AXIS

### Molecular Overview

**Figure 1:**
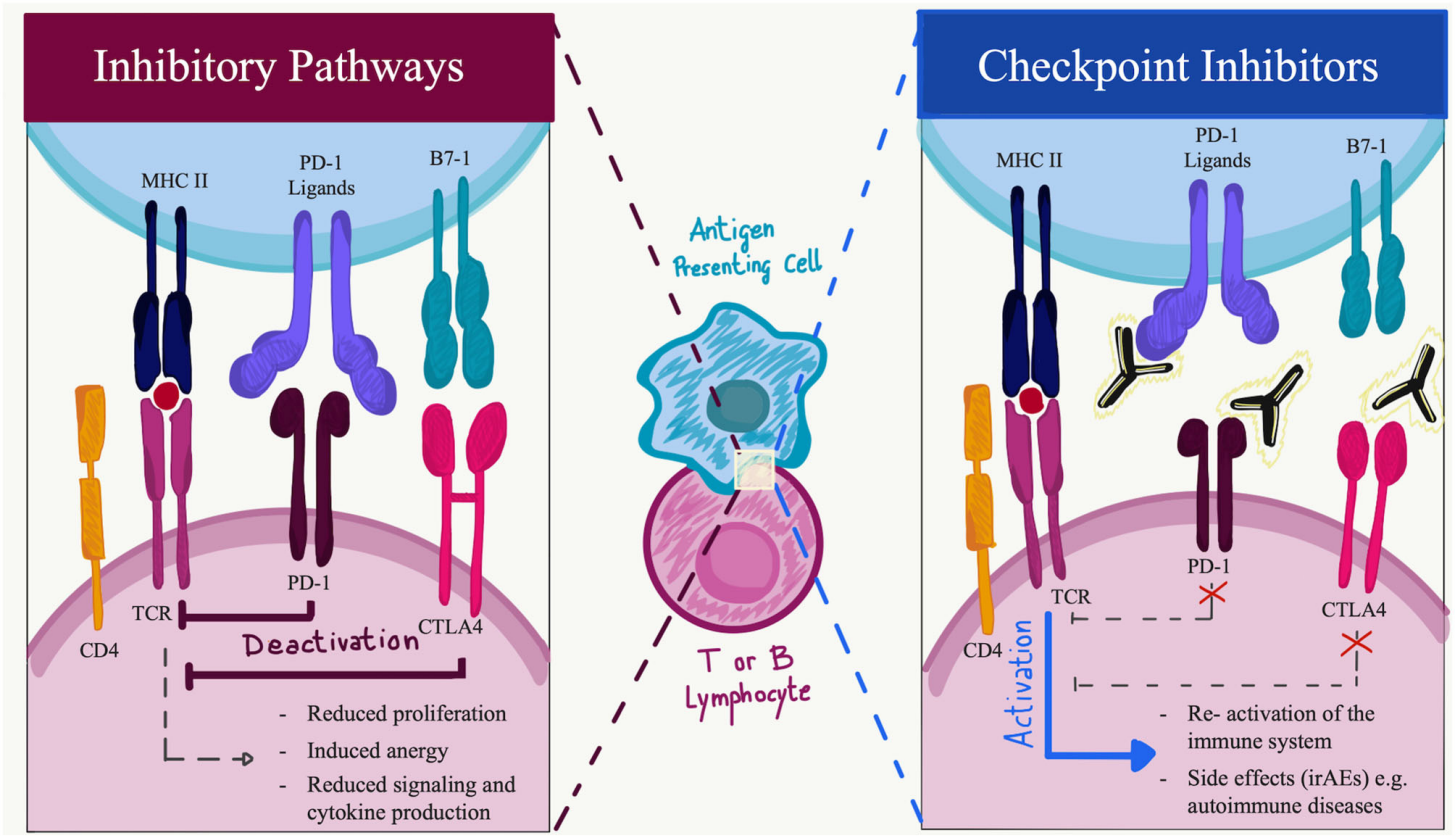
Immune checkpoint function during activation or inhibition pathways. Made with Adobe Draw.

Programmed cell death protein-1 is a 288 amino acid protein that belongs to the immunoglobulin superfamily and is a homolog to CD28. PD-1 is expressed in physiological conditions on a subset of thymocytes but can be induced upon activation in many types of immune cells, including T cells, B cells, natural killer (NK) cells, monocytes, and dendritic cells (DCs) (5). In its cytoplasmic tail, PD-1 has two tyrosine motifs, an immunoreceptor tyrosine-based switch-motif (ITSM) and an immunoreceptor tyrosine-based inhibitions motif (ITIM) (7). PD ligands are members of the B7 family of type 1 transmembrane proteins, which also include CD86 and CD80 (8). They have similar exon organization of the 5′UTR region, a signal sequence, IgV-like, IgC-like, and transmembrane domains, cytoplasmic exon 1, and cytoplasmic exon 2 with the 3′ untranslated region (8). However, the affinity of PD-L2 to PD-1 is three times stronger when compared to that of PD-L1, and this is probably due to tryptophan that is unique to PD-L2 (9). PD-1 ligands differ in their expression patterns: PD-L2 expression is restricted to professional antigen-presenting cells (APCs) (10), while PD-L1 is ubiquitously expressed in the inflamed tissues (11). To date, in physiological conditions, PD-L1 mRNA is largely present in various tissues, while PD-L1 protein is barely expressed on the cell surface, suggesting that PD-L1 mRNA is under tight post-transcriptional regulation. An exception is made in the context of human cancers, where PD-L1 protein is highly expressed by the tumor cells in an attempt to hide neoantigens from immune surveillance (12). The engagement of PD-1 by its ligands leads to the formation of PD-1 microclusters together with the T-cell or B-cell receptor (TCR or BCR). This leads to the recruitment of the Src homology phosphatase (SH)-2 domain-containing tyrosine phosphatase 2 (SHP2). Which then causes a decrease in the phosphorylation of the entire spectrum of TCR downstream signaling molecules. PD-1 engagement decreases the downstream signaling of both T- and B-cell receptors, respectively, by decreasing the phosphorylation of CD3z and protein kinase C q (PCK-q) and that of Igb, Syk, and phospholipase Cg2 (PLCg2). Furthermore, PD-1 engagement leads to the blockage of both the phosphatidylinositol-3 kinase and the serine-threonine kinase Akt through the recruitment of SHP2 (13). The downstream effects of PD-1 and PD-L1/L2 interaction comprehensively result in reduced proliferation of autoreactive T cells, suppression of effector T and B cells in parenchymal tissues, reduced cytokine production, induced T-cell anergy and exhaustion, reduced motility, and increased IL-10 production (14). The absence of PD-1 leads to an alteration of the signaling threshold during the development of T cells in the thymus, resulting in an increased presence of CD4/CD8 double-negative T cells. Furthermore, in several preclinical models, the blockade of the PD-1 pathway results in the development or exacerbation of autoimmune diseases depending on the genetic background they have (15–17). For example, C57BL/6 PD-1^−/−^ mice develop lupus-like glomerulonephritis and arthritis starting at 6 months of age, while BALB/c knockout mice develop a dilated cardiomyopathy starting at 5 weeks of age (18–20). Among others, these findings suggest that the PD-1 axis plays an important role in central and peripheral tolerance, and a preventive role for several types of autoimmune disorders (Figure 1).

### PD-L1 and PD-L2 Expression in the CNS During Neuroinflammation

Preliminary evidence of the expression of PD-1 ligands in the central nervous system (CNS) comes from experimental autoimmune encephalomyelitis (EAE). In EAE mice, PD-L1 is overexpressed on microglial cells, astrocytes, and infiltrating mononuclear cells near the meninges, particularly in the areas with the highest inflammatory response (21). PD-L1 expression is also increased on the endothelium surrounding the cell infiltrates. Microglial cells, which represent 5–20% of all glial cells in the murine CNS, constitutively express a low level of PD-L1, and its expression can be upregulated *in vitro* when exposed to inflammatory conditions, for example, in the presence of IFN-γ or Th1 supernatants. PD-L1 expression by microglia can regulate immune responses by interacting with PD-1. Thus, one of the current hypotheses is that PD-L1 is expressed by microglia and infiltrating cells, which might act a strong immune inhibitory molecule that is able to curb T-cell activation and thus useful to maintain immune homeostasis in the CNS (11, 22).

The functions and distribution of PD-L2 are similar but not overlapping to PD-L1, and they still need to be fully elucidated in the CNS. Similar to PD-L1, PD-L2 inhibits T-cell proliferation by blocking cell cycle progression without increasing cell death. However, PD-L2 seems to be slightly less potent than PD-L1. Moreover, PD-L2 seems to be upregulated on small round cells in the brain, indicative of infiltrating macrophages or B cells (11).

To date, some authors reported that PD-L2 might bind to a second receptor different from PD-1, and is known as repulsive guidance molecule b (RGMb). RGMb, also called DRAGON, is a part of the RGM family, a group of glycosylphosphatidylinositol-anchored membrane proteins that bind bone morphogenetic proteins (BMPs) and neogenin. RGMb does not directly act as a signaling molecule, although it can function as a co-receptor modulating BMP signaling. RGMb is expressed mainly in the CNS and in particular on the surface of macrophages and other immune system cells. There is some evidence that the interaction of PD-L2 with RGMb through the BMP pathway might be co-stimulatory rather than inhibitory on T cells (23). This interaction seems to promote the development of respiratory tolerance, facilitating the proliferation of T cells. However, the potential role of RGMb has just begun to emerge, and further studies are needed to clarify its functions.

## PD-1 Role During Neuroinflammation

The multifactorial nature of neuroinflammation unfolds through a complex, highly multicellular pathophysiological process that evolves according to the type and the duration of the disease. Many studies have tried to explain the role of the PD-1/PD-L1 pathway in CNS infections, often with contradictory results. In this section, we will discuss recent findings from various studies in murine models, focusing on the different cellular subsets that are involved (Table 1).

**Table 1:** PD-1 and its ligands in animal models of neurological disorders.

**Disease**	**Model**	**Cell subset involved in the axis**		**Reference**
		**PD-1**	**PD-L**	
Viral infection	Viral encephalitis: WT and PD-1 KO MuPyV infected mice	CD8^+^ bT_RM_ cells	Microglia, astrocytes and infiltrating monocytes	Shwetank et al. (24)
	Chronic neuroinflammation: MCMV infected mice	CD8^+^ T cells	Microglia, astrocytes	Schachtele et al. (25)
	Chronic neuroinflammation: WT and PD-1 or PD-L1 KO MCMV infected mice	CD8^+^ bT_RM_ cells	?	Prasad et al. (26)
	Chronic murine retroviral (LP-BM5) infection (WT and PD-1 KO mice)	?	Microglia	Chauhan et al. (27)
Multiple Sclerosis	EAE mice	CD4^+^ T cells	?	Mair et al. (28)
	EAE mice injected with MIS416, a TLR9 and NOD2 agonist	?	Neutrophils, macrophages and infiltrating monocytes	White et al. (29)
	EAE mice injected with MIS416	?	Neutrophils	Khorooshi et al. (30)
	EAE mice	CD4^+^ T cells and microglia	Microglia	Hu et al. (31)
	EAE mice with a transfer of granulocytic myeloid-derived suppressor cells (G-MDSCs)	T cells	G-MDSCs	Ioannou et al. (32)
Parkinson's Disease	Parkinson's disease mouse model and PD-1 KO mice	Iba1^+^ microglial cells	?	Cheng et al. (33)
Spinal cord injury	Mice with mid-thoracic spinal cord injury	CD8^+^ T cells	Macrophages	Norden et al. (34)
	Murine primal cord injury model using T-and-B-cell-deficient Rag1-/- mice	Tregs	Macrophages, microglia	He et al. (35)
	Rat spinal cord injury model (Dexmedetomidine administration)	Microglia	?	He et al. (36)
Tumor	GL261 murine glioma model	CD4^+^ and CD8^+^ T cells	Microglia and macrophages	Qian et al. (37)
	PCNSL cell lines and human monocyte-derived macrophages	?	Microglia and macrophages	Miyasato et al. (38)
Intracerebral hemorrhage	PD-1 KO and WT ICH mice	Macrophages	?	Yuan et al. (39)
Surgical Brain Injury	SBI mouse model (PD-L1 mAb/PD-L1 protein administration)	?	Microglia	Chen et al. (40)
Chronic neurodegeneration	Murine prion disease model-ME7 strain (PD-1 KO and WT mice)	Microglia	Neurons	Obst et al. (41)
Traumatic brain injury	Controlled cortical impact model of traumatic brain injury	T cells	Astrocytes	Gao X et al. (42)

### Lymphoid Cells

As highlighted in the Introduction section, PD-1 acts as an important immunosuppressive molecule that can exert several regulatory roles on T-cell effector functions. It has extensively been demonstrated that signaling through the PD-1 receptor results in the inhibition of TCR-mediated cell activation (e.g., proliferation and cytokine production), (8, 43, 44) but increasing evidence suggests that the PD-1 axis also has a crucial role in modulating T-cell activation in different models of neuroinflammatory diseases.

#### CD4 and CD8 T Lymphocytes

Mair et al. studied the role of PD-1 in the development and maintenance of T-cell adaptive tolerance [i.e., a process by which T cells acquire a hyporesponsive state when antigen stimulation persists *in vivo* (28)] after exposure to high levels of autoantigens in the model of EAE. This group reported that PD-1 expression is upregulated on adapted CD4^+^ T cells within the CNS, but PD-1 loss did not preclude T cells to acquire an adapted phenotype both *in vivo* and *in vitro*, indicating that the maintenance of their unresponsiveness in EAE is independent of PD-1 (28). This finding suggests that PD-l is not fundamental in this process, and it is in contrast to the majority of studies about PD-1 involvement in T-cell adapted state (45). Using the same disease mouse model (i.e., EAE), it was shown that CD4^+^ T cells, infiltrating the CNS at the peak of disease, upregulate PD-1, and this overexpression is associated with a reduction of Th1 cells due to nitric oxide release by PD-L1^+^ microglia. This evidence supports the role of PD-1 in EAE regulation through the suppression of Th1 cell differentiation (31).

The impairment of CD8^+^ T-cell function found in mice with spinal cord injury (SCI) was associated with the induction of regulatory pathways, including the upregulation of PD-1/PD-L1. Injured mice display an increased percentage of PD-1^+^ CD8 T cells which also have a higher expression of this receptor on their surface. In this way, PD-1 upregulation prevents the immune-inflammatory cascade and limits the spreading of inflammation at the site of damage (34).

Using the same mouse model, the study of He et al. showed that the anti-inflammatory function carried out by regulatory T cells (Tregs) that infiltrate the spinal cord in the SCI subacute phase is maintained, thanks to the overexpression of PD-1. Knockdown of PD-1 in Tregs caused decreased production of IL-10, TGF-β, and Foxp3, inducing a lower inhibitory activity of Tregs on pro-inflammatory macrophages/microglia (35).

In the glioma 261 model (GL261), through the analysis of cells that infiltrate the glioma, Qian et al. demonstrated that PD-1 expression is higher both on CD4^+^ and CD8^+^ T cells and increases during tumor progression. Besides, PD-1 expression, in cells that infiltrate the tumor, correlates with an increased apoptotic rate of T cells, suggesting a role for the PD-1 axis in the inhibition of T-cell function in glioma (37). Some evidence regarding the importance of the PD-1/PD-L1 pathway in controlling neuroinflammation also comes from different models of chronic neuroinflammation in mice recovering from a viral brain infection.

In mice with encephalitis induced by murine cytomegalovirus (MCMV) infection, expression of PD-1 is found on CD8^+^ T cells within the brain. Such expression contributes to their functional suppression and cytokine production inhibition in the post-encephalitic brain (25).

#### Brain-Resident Memory T Cells (BT_RM_s)

In 2017, Qian et al. assessed the importance of the PD-1 axis in the generation of brain-resident memory T cells (bT_RM_s) after viral infection. bT_RM_s are a population of tissue-resident CD8^+^ lymphocytes that persist for a long time in the brain and play a fundamental role in controlling pathogen clearance in the case of virus re-infection (37, 46).

Authors phenotypically characterize bT_RM_s by analyzing CD8^+^ lymphocytes residing in the brain of mice chronically infected with MCMV and describe that loss of PD-1 results in fewer bT_RM_ cells within the brains of PD-1 KO mice (i.e., the reduced number of CD8^+^ cells expressing the integrin CD103, a marker of brain T_RM_). Furthermore, the characterization of PD-1 expression on CD103^+^CD8^+^ T cells isolated from the brain at 30 days post-infection reveals a higher frequency of PD-1^+^CD103^+^ cells in the brains of wild-type animals compared to PD-L1 KO mice. These results suggest that the upregulation of PD-1 receptors on bT_RM_ cells may help to preserve their longevity (26). Moreover, Shwetank et al. confirmed that PD-1 signaling plays a pivotal role in the regulation of immune response in CNS persistent infection, revealing a multifaceted function of PD-1 on neuroinflammation regulation in mouse polyomavirus (MuPyV) encephalitis. These authors demonstrate that the intracerebral inoculation of MuPyV leads to the generation of a permanent population of virus-specific PD-1^+^bT_RM_ cells in infected mice brains. During the acute phase of infection, PD-1 inhibits the effector functions of virus-specific CD8^+^ bT_RM_ cells, limiting the severity of neuroinflammation but maintaining the control of re-encountered virus during persistent infection (24).

All these findings suggest that PD-1 signaling represents not only a mechanism to inhibit T-cell stimulation and proliferation but also to promote the long life of bT_RM_ cells.

Evidence supporting the role of the PD-1 regulator in bT_RM_ cells also comes from studies conducted in the human brain. In line with results obtained in mouse models, Smolders et al. demonstrated that in human white matter CD103^+^ CD8^+^ T_RM_ cells, PD-1 is highly expressed (47) and that this cell population is enriched in the active lesions in multiple sclerosis (48).

### Myeloid Cells

Both peripheral and CNS-resident myeloid cells are remarkably involved in the onset and development of neurological disorders. The pathogenesis of many neuroinflammatory diseases has been associated with impaired neuronal function due to the production of reactive oxygen species (ROS) or reactive nitrogen species (RNS). This consequently leads to an increase in oxidative stress (49). In mice, GM-CSF, IFNγ, and IL1ß are fundamental stimulations for the recruitment of myeloid cells in the CNS (50–53). Accordingly, receptors for IFNγ and GM-CSF are abundant on a variety of myeloid cells, such as neutrophils, DCs, macrophages, and monocytes (54). It has been demonstrated that GM-CSF plays a fundamental and non-redundant role in EAE pathology (55). IL1ß is involved both in the development of EAE and in the transmigration of myeloid cells into the CNS (56). Moreover, endothelial cells activated with IL1ß release GM-CSF which then converts monocytes into antigen-presenting cells (APCs). Mice depleted for both IL1ß and GM-CSF are resistant to EAE (57), highlighting the importance of myeloid cell recruitment in the CNS. On the same note, IFNγ seems to be fundamental for the gradual acquisition of a mature inflammatory phagocyte phenotype in Ly6C^hi^ monocytes (50). Only in recent years, an increasing number of studies have highlighted the involvement of myeloid cells expressing PD-1/PD-L in the immune regulation of CNS disorders in different contexts. Ioannou et al. showed that granulocytic myeloid-derived suppressor cells (MDSC) expressing PD-L1 could be found in the peripheral lymphoid tissue of EAE mice. PD-L1 upregulation was stimulated by IFNγ, and the active transfer of these cells was able to ameliorate the disease, significantly decrease demyelination, and delay the onset of symptoms (32). As an example, studies conducted with MIS416, a small molecule that is a TLR9 and NOD2 agonist, show interesting results in the EAE animal model. MIS416 treatment produced an expansion of myeloid cells and an increased expression of PD-L1 on the peripheral myeloid subset (CD11b^+^, CD45^high^) that was recruited to the CNS (29). Moreover, MIS416 has shown success in reducing disease burden in mice with EAE when administered prophylactically or therapeutically. This protection depends on the rapid production of IFNγ, a cytokine that was also significantly increased in MIS416-treated secondary progressive MS patients (58). MIS416 leads to an upregulation of PD-L1 and MHCII on neutrophils, macrophages, and infiltrating monocytes and enhances the homeostatic recruitment of PD-L1-expressing myeloid cells to the CNS (30).

#### Monocytes and Macrophages

Infiltrating monocytes and monocyte-derived macrophages are fundamental for the pathogenesis and the development of inflammation inside the CNS. Differentiated inflammatory monocytes and their progeny are increasingly present in the inflamed tissue and represent the most important executors of GM-CSF-dependent pathogenesis (54). In 2019, Schwartz's group showed that immune checkpoint blockade targeting the PD-1/PD-L1 pathway might have beneficial results in a tau-driven disease model resembling Alzheimer's disease (AD). The use of PD-L1 blocking antibodies resulted in increased immunomodulatory monocyte-derived macrophages within the brain parenchyma, which ultimately led to the modification of brain pathology and the restoration of cognitive performance (59, 60).

Yuan et al. conducted a study exploiting an animal model of intracerebral hemorrhage (ICH), which accounts for 10–15% of all acute strokes, and showed that PD-1 expression is increased in perihematomal tissue and mainly on CD11b^+^ macrophages. The expression of PD-1 attenuates macrophage-mediated inflammation and brain injuries (39).

On the other hand, in an animal model of spinal cord injury, Norden's group showed that macrophages were highly activated in the spleen of mice, with an increased expression of PD-L1 and MHCII after the induction of the disease (34).

#### Neutrophils

The importance of neutrophils in neuroinflammatory disorders is gaining growing interest, and their involvement in the pathogenesis and progression of a stroke, MS, and Alzheimer's disease is now undeniable (61–64). With their capability to release ROS, enzymes, neutrophil extracellular traps (NETs), and cytokines in different pathophysiological conditions, neutrophils are involved not only in acute inflammation but also in causing chronic collateral damage in tissues (65). However, several studies reported a duality in the function of neutrophils, as they carry an active contribution both in a pro-inflammatory and an anti-inflammatory way. On the one end, neutrophils perform several crucial functions during all the stages of autoimmune progression, including antigen presentation, modulation of several cell types, and direct tissue damage (66). In human patients, the neutrophil-to-lymphocyte ratio has been proposed as a clinical marker both for MS and ischemic stroke (67). Accordingly, the percentage of neutrophils in the infiltrating cells in EAE, MS preclinical model, increased remarkably during the disease onset, remaining high until the peak stage and then drastically decreasing thereafter (68). Moreover, blocking a fundamental cytokine for the recruitment of neutrophils, namely CXCR2 (CXC motif chemokine receptor 2), resulted in reduced disease severity in EAE (69). On the other hand, several recent papers reported that neutrophils might exert a protective function in neuroinflammatory disorders, by slowing down the disease progression (70). On the same note, Khorooshi et al. reported that MIS416 injected intrathecally in EAE mice suppressed the disease and recruited neutrophils into the CNS. In the first phase of the EAE, these neutrophils were described as “protective,” as they were able to produce IL-10 and express PD-L1. When tested for *in vitro* proliferation assays, PD-L1^+^ neutrophils showed immunosuppressive features, slowing down lymphocyte proliferation, while PD-L1^+^ monocytes cannot (30). Moreover, Melero-Jerez et al. identified myeloid-derived suppressor cells expressing Arg1^+^PD-L1^+^ and Gr1^+^. These cells were induced after IFN-ß treatment and once again showed immunosuppressive features toward T lymphocytes (71).

#### Microglia

Microglial cells are CNS-resident myeloid cells that are extremely sensitive to alterations in the homeostasis of the brain. In autoimmune inflammation, microglia act as a direct link between the immune system and the CNS (21). Microglial cells express basal levels of PD-L1 (approximately 20%) in uninfected mice, but this expression can be increased to over 90% of the cells within 1 week following a viral brain infection (25). In 2018, Chauhan et al. exploited a model of chronic murine retroviral (LP-BM5) infection. They described that the infiltration rate of T lymphocytes and macrophages, as well as microglial activation, was remarkably increased in PD-1 KO mice compared to wild-type animals (27). A basal level of PD-L1 protein expression was observed on approximately 20% of microglial cells in uninfected mice, but it reached over 90% of the cells within 7 days following viral brain infection. In MCMV-induced brain infection, there is chronic neuroinflammation and the production of IFNγ by infiltrating T lymphocytes, which induces the upregulation of PD-L1 in activated brain-resident glial cells. The antiviral responses are controlled through functional inhibition of effector CD8^+^ T cells via the PD-L1 pathway, limiting the consequences of neuroinflammation (25). During neuroinflammation, both microglial cells and astrocytes upregulate PD-L1 along with MHC I and MHC II, suggesting a role for resident glial cells in limiting CNS pathology (72). PD-L1 expression on microglia and astrocytes, but not on oligodendrocytes, has also been reported to inhibit T-cell activation and limit immune-mediated tissue damage in a mouse model of acute viral encephalitis (MuPyV encephalitis). PD-1 acts to inhibit the effector functions of virus-specific CD8^+^ bTRM during MuPyV encephalitis (24). This study highlights the role of PD-L1 in mediating protection from viral-induced immunopathology associated with encephalomyelitis.

Several other studies with murine models illustrate the immunoregulatory role of microglial cells during chronic, persistent neuroinflammation. At the peak of the disease in the EAE model, Hu et al. found that microglial cells were increased in number and displayed an upregulation of PD-L1 along with MHC II and CD86. Using an *ex vivo* co-culture, microglia from EAE mice inhibited antigen-specific CD4^+^ T-cell proliferation, as well as Th1 differentiation via nitric oxide (whose production was dependent on PD-L1) (31).

Moreover, the adoptive transfer of M2-polarized microglia expressing PD-L1 attenuated the severity of an established EAE, demonstrating again the importance of the regulatory role of PD-L1 in neuroinflammation (73).

In a murine model of chronic neurodegeneration, a prion disease in the ME7 strain, PD-1 expression was increased on microglia cells compared to wild-type mice. Moreover, PD-1^−/−^ mice did not show an increase in myeloid cell infiltration or a major change in the inflammatory profile. Furthermore, no changes were observed in the neurodegeneration of the pyramidal neurons in the hippocampus that indifferently expresses PD-L1 and PD-L2 both in healthy controls and in ME7 mice. The absence of PD-1 led only to a slight exacerbation in the behavioral phenotypes (41).

The involvement of the PD-1 axis has also been studied in a model of surgical brain injury (SBI). PD-L1 was significantly upregulated on microglial cells both *in vitro* and *in vivo* through the ERK signaling pathway. Consistently, the blockade of the PD-1 axis using a PD-L1 antibody significantly enhanced brain edema, exacerbated apoptosis, and increased neurodeficits post-SBI. On the other hand, the activation of PD-1/PD-L1 signaling with a PD-L1 recombinant protein significantly attenuated the inflammatory responses and brain edema post-SBI. Thus, the PD-1/PD-L1 pathway might be involved in a “self-protection mechanism” in SBI (40).

Dexmedetomidine, a drug used in a rat SCI model, inhibits neuroinflammation through the upregulation of PD-1 on microglia cells mediated by AMPK signaling. PD-1/PD-L1 interactions downregulate pro-inflammatory cytokine expression by activating microglia and inducing the M2 polarization of microglial cells (36). Besides suppressing T-cell activity, the PD-1/PD-L1 axis might also prevent the immune system from eliminating cancer cells. The role of PD-L1 on brain tumors might serve as an immune evasion strategy. Miyasato et al. reported that PD-L1 expression was upregulated on tumor-associated macrophages/microglia in the case of primary central nervous system lymphoma (38). In 2017, Saha et al. demonstrated that the triple-combination therapy of anti-cytotoxic T-lymphocyte-associated protein (CTLA)-4, anti-PD-1, and G47Δ-mIL12 (oncolytic HSV expressing murine IL-12) healed most mice in two glioma models. This curative effect was associated with macrophage infiltration and the subsequent M1-like polarization, along with an increase in the ratio of T-effector to T-regulatory cells (74). Immune checkpoint inhibitors function as tumor-regressing factors via the modulation of the interactions between immune and tumor cells. They lead to the reactivation of cytotoxic T cells which fight against cancer cells. Moreover, in 2018, Qian et al. identified the distribution of tumor-infiltrating T cells and PD-L1 expression in a model of murine glioma. The group showed that the IFNγ level was positively correlated with PD-L1 expression in the glioma microenvironment (37). Finally, in a preclinical model of Parkinson's disease, Cheng et al. hypothesized the involvement of the PD-1/PD-L axis in the pathogenesis of the disease. The knockout of PD-1 led to an exacerbation in the motor dysfunction of animals. This was explained by an increase in microglial activation and release of pro-inflammatory cytokines, which ultimately led to neuroinflammation in midbrains (33).

#### Astrocytes

Astrocytes are a very heterogeneous population in the CNS. Similar to microglia, they can switch to a reactive state and can modulate the progression of multiple neurologic disorders both positively and negatively (75). Similar to microglia, during neuroinflammation, astrocytes can upregulate PD-L1 along with MHC I and MHC II, suggesting a role for resident glial cells in limiting CNS pathology (72). Accordingly, Lipp et al. showed an upregulation of PD-L1 on hippocampal astrocytes in a model of axonal degeneration (76), suggesting a potential role in the inhibition of T cells. Gao et al. described the formation of dense areas of activated astrocytes expressing PD-L1 near the lesions in a traumatic brain injury model (42). In this model, astrocytes acted as gatekeepers, by blocking the infiltration of inflammatory Ly6c^hi^ monocytes/macrophages, but not the entrance of tissue repairing Ly6c^low^F4/80 (42) cells.

## PD-1 Pathways in Human Neuroinflammatory Disorders

### Immune-Related Adverse Effects of Checkpoint Inhibitors

Due to the high level of expression of PD-L1, monoclonal antibodies targeting the PD-1 axis have been developed to treat a wide variety of human tumors. Nivolumab was the first PD-1 checkpoint inhibitor approved for the treatment of metastatic melanoma (77). The approval of these drugs improved the treatment of several types of cancer. To date, the immune checkpoint blockade approach is generally safer than common chemotherapy. On the other hand, the possible side effects on the immune system are not completely understood, and they have been associated with irAEs. These side effects mostly involve compartments like the gastrointestinal tract, endocrine glands, skin, liver, and the CNS. Neurological autoimmune diseases are reported within the irAEs in the clinical trials, but at a low frequency (78). During post-marketing surveillance, neurological complications have been described with an estimated incidence of about 3–4% (3, 4). Some authors reported neuromuscular complications as the most common nervous system condition, (3) but cases of multiple sclerosis and meningoencephalitis have been increasingly reported in the treated patients (79, 80). The clinical management of neurological adverse events is not trivial, which can be attributed to the partial knowledge of the underlying immunological mechanisms (81). Systemic corticosteroid therapy is the most common first-line therapy for irAEs (82). Clear guidelines for the treatment of neurological adverse events are still missing. Typically, drugs interfering with the PD-1 axis have been associated with irAEs at a lower frequency compared to those binding other immune checkpoint inhibitors (i.e., CTLA-4). The risk rises when these therapies are combined (83). Neurological irAEs seem to show a different trend with an overall incidence of 6.1% in patients treated with anti-PD-1 antibodies, 3.8% with anti-CTLA-4 antibodies, and 12% in combination (84). Diamanti et al. recently reported common neurological manifestations of irAEs in a small cohort of 27 oncologic patients, including myositis, inflammatory polyradiculoneuropathies, and myasthenia gravis, alone or in combination. The authors suggest that in corticoresistant or severely affected patients, second-line treatments with IVIg or plasmapheresis may provide additional benefit (85).

### PD-1 and Multiple Sclerosis

The immune-regulatory role of PD-1 in MS, the most common neuroinflammatory disease, is still to be fully elucidated. However, studies have demonstrated the importance of the PD-1 pathway in the development and progression of EAE, suggesting that this pathway might play a role in human disease as well. In people with MS, the PD-1 gene polymorphism (PD-1.3), which is related to reduced PD-1 activity, was associated with a progressive course of the disease, possibly due to a partial defect in PD-1–mediated inhibition of T-cell activation (86). Pawlak-Adamska et al. in a population-based case-control study with 203 MS patients investigated and selected four PD-1 single-nucleotide polymorphisms: rs36084323 (PD-1.1), rs11568821 (PD-1.3), rs2227981 (PD-1.5), and rs2227982 (PD-1.9). The study revealed that the polymorphic variations could be rather disease-modifying than MS risk factors (87).

The relative expression of PD-1 and PD-L1 in the PBMCs of MS patients seems to be significantly decreased compared to healthy donors (88). Javan et al. showed a general reduction in the expression of inhibitory receptors like PD-1, CTLA-4, and TIM-3 in the PBMCs of MS patients, particularly for PD-1 (89). Moreover, after treatment with autologous hematopoietic stem cell transplant in MS patients, Arruda et al. observed a temporary increase in the number of regulatory T cells and PD-1- expressing CD8^+^ T cells (90). The expansion of CD8^+^PD-1^+^ T and CD19^+^PD-1^+^ B cells was associated with better clinical outcomes. Interferon-beta, a primary immunomodulatory treatment for MS, enhances PD-L1 expression *in vitro* as well as *in vivo* on APCs (91). Koto et al. highlighted differences in the presence of circulating CD8^+^ PD-1^+^ T cells according to the stage of the disease. In fact, in the disease remission state, CD8^+^ PD-1^+^ T cells were decreased in the peripheral blood of patients with MS and resolved in patients treated with IFN-β treatment who showed immune-regulatory cytokine interleukin (IL)-10 expression. On the other hand, CD8^+^ PD-1^+^ T cells were enriched in the CSF of MS patients, which predicted a good response to subsequent IV steroid therapy (92).

Regarding the neuropathological analysis of post-mortem brain tissues, Pittet et al. showed that PD-L1 is largely expressed in MS lesions compared to controls and that it is colocalized with astrocytes or microglia/macrophage markers. On the contrary, PD-L2 expression was notably reduced on brain endothelial cells of MS brains, while being easily detectable in controls. In this case, only a small number of infiltrating CD8^+^ T lymphocytes in the lesions expressed PD-1 (93). One possible explanation is that during MS pathogenesis, the inflamed CNS attempts to protect itself against active T lymphocytes through the expression of PD-L1. However, this process is not effective since the majority of CD8^+^ T infiltrating lymphocytes lack PD-1 and are insensitive to PD-L1/L2 (93). On a different note, van Nierop et al. reported that post-mortem brains of patients with an advanced disease contained a high frequency of CD8^+^ T cells that expressed both co-inhibitory (TIM-3 and PD-1) and co-stimulatory (ICOS) T-cell receptors (94).

The usage of checkpoint inhibitors in clinics (such as ipilimumab, an anti-CTLA-4 antibody) was associated with MS development and an increase in MS activity (95). Other checkpoint inhibitors like nivolumab, ipilimumab, pembrolizumab, and atezolizumab were associated with MS relapse (80, 96). A recent meta-analysis described a rapid progression of MS in 14 patients with MS and concomitant immunotherapy (97). Gerdes et al., with quantitative NGS, showed that distinct clonal expansions of CD4^+^ and CD8^+^ T cells in the melanoma and CSF were found during ipilimumab treatment, and concomitant MS activity permitted conversion of RIS to MS (97). These data suggest that the protective antitumor response could be associated with inadvertent anti-CNS autoimmune response toward different antigens and MS reactivation (91).

### PD-1 and Alzheimer's Disease

Alzheimer's disease is the most common cause of dementia in humans and is characterized by a decline in cognitive decline and neuronal loss. AD pathologies are characterized by two conditions: β-amyloid (Aβ)-containing extracellular plaques and tau-containing intracellular neurofibrillary tangles (98). The mechanism that underlines the deposition of β-amyloid and TAU protein remains indecipherable, and the immune system may play an essential role in the pathological process (99). Recent evidence suggests that systemic immunity should be boosted, rather than suppressed, to promote brain repair through an immune-dependent cascade. In this context, the PD-1 pathway has been proposed as a possible target therapy for Alzheimer's disease (100). In mouse models of AD, trafficking of blood-borne myeloid cells (monocyte-derived macrophages) to the CNS was shown to be neuroprotective. The expression of both PD-1 on T cells and PD-L1 on monocytes and macrophages decreases significantly in AD patients and patients with mild cognitive impairment compared to age and sex-matched healthy controls (101). Baruch et al. demonstrated that PD-1 blockade evokes a systemic IFN-γ-dependent immune response that enables the mobilization of monocyte-derived macrophages into the brain (100). This process is reminiscent of tissue-specific immune surveillance induced by ICI blockade in cancer therapy. Moreover, PD-1 signaling impairments inhibit IL-10 production, suggesting that positive PD-1 signaling increases IL-10 production. IL-10, in turn, can reduce inflammatory responses and ameliorate AD pathology as demonstrated in animal models (102).

Besides modulating peripheral blood cells, this route may also influence the resident cells of CNS like microglia and astrocytes. Recent work also showed that PD-L1 was increased in the CSF of AD patients (6). PD-L1 expression in astrocytes and PD-1 expression in microglia are close to amyloid plaques in AD patients and AD animal models. Kummer et al. demonstrated that PD-1/PD-L1 signaling is an important factor in Ab phagocytosis, with PD-1 knockout resulting in increased Ab levels, amyloid plaques, and cognitive deficits in APP/PS1 mice together with an inflammatory response in PD-1-deficient microglia (103). Instead, Latta-Mahieu et al. showed that inhibition of PD-1 checkpoint signaling in some different animal models by itself is not sufficient to reduce amyloid pathology (104). In conclusion, the PD-1/PD-L1 axis is a promising target for AD treatment but will need to be further examined to translate the data from animal models to clinical trials.

## Discussion

In basic research, the PD-1/PD-L axis has been identified as being of primary importance to immunosurveillance. Studies in preclinical models suggest that this axis plays an important role in different pathological conditions involving the CNS, such as viral encephalitis, brain tumors, autoimmune disorders, and dementia. In most cases, the authors describe an upregulation of PD-1 or PD-Ls during the pathological condition. PD-L1 and PD-L2 seem to be expressed and upregulated mainly on myeloid-derived cells, namely, resident microglial cells and infiltrating macrophages and neutrophils. Conversely, the receptor PD-1 is predominantly found on T and B lymphoid cells, although there is some evidence that it can be upregulated also on activated microglia and macrophages. Under the current understanding of the PD-1/PD-L1 axis in the CNS, its role cannot be described uniquely as protective or pathogenic. In most cases, the upregulation of PD-L1 or PD-L2 helps in slowing down and limiting the inflammatory process, suggesting a protective mechanism (40, 73). However, the use of PD-L1 blocking antibodies might have beneficial results in other contexts, such as in a tau-driven disease model resembling Alzheimer's disease, as described by Schwartz's group (59). Moreover, in the first attempt at a therapeutic approach, Hirata et al. (105) transferred genetically modified dendritic cells presenting MOG peptide in the context of MHC class II and overexpressing PD-L1 before the EAE induction. This treatment reduced T-cell response to MOG, cell infiltration into the spinal cord, and ultimately the severity of the disease. Moreover, the administration of αPD-1 antibody that possesses selective toxicity both *in vitro* and *in vivo* for PD-1^+^ cells restored mobility in mice that were paralyzed by EAE (37, 106). Most of the current knowledge about neurological pathologies is based on studies of mice. Since murine models cannot reliably reproduce the complexity of pathologies observed in the central nervous system, it is widely recognized that this is a limitation in the understanding of neurological disorders. At the moment, only a few studies focused on neurological patients and with controversial results (89, 107).

**Figure 2:**
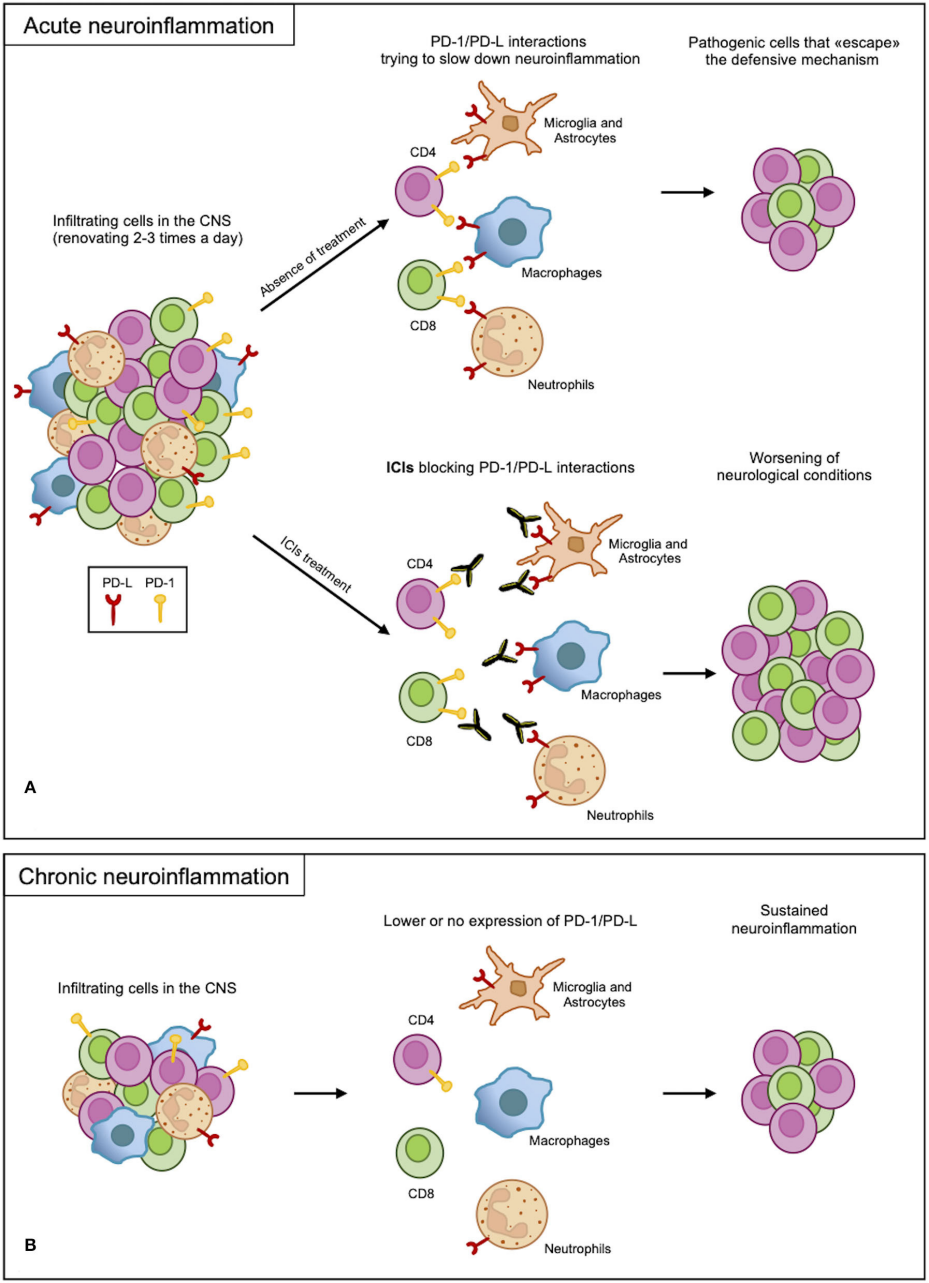
PD-1/PD-L axis in neuroinflammation. **(A)** In acute neuroinflammation, CNS infiltrates are renewed 2-3 times a day. Infiltrating myeloid and resident cells expressing PD-Ls attempt to slow down the inflammatory process through interactions with PD-1 expressed on the lymphoid cells. Due to an overabundance of cells, some pathogenic cells escape this protective mechanism, causing damage. However, blocking PD-1/PD-L interactions with ICI treatments remove this defense, leading to the worsening of the inflammation. **(B)** In chronic inflammation, the PD-1/PD-L axis may not function properly as a result of low or no expression of the receptor and ligand; therefore, the sustained inflammation continues.

From the presented results, the PD-1/PD-L axis appears to be regulating the immune response but is not involved in determining the disease or in causing exacerbation. Indeed, inhibition of this axis increases the severity of neuroinflammation, which occurs as a side effect of PD-1 axis inhibition in cancer (Figure 2A). Most results, however, rely on mouse models of acute inflammation and indicate the upregulation of the PD-1/PD-L axis as a counteracting mechanism trying to re-establish homeostasis. Indeed, anti-migratory therapies, diminishing the number of blood-derived CSN-infiltrating cells, are very efficacious in MS. This suggests that inhibitory checkpoints, including the PD-1 axis, can take care of the few residual inflammatory cells still infiltrating the CNS. On the other hand, if the PD-1/PD-L axis fails in the long term, its contribution to chronic CNS inflammation leading to neurodegeneration is not currently known (Figure 2B). The latter hypothesis, if confirmed, would highlight a potential therapeutic strategy in fostering, supporting, and reinforcing this axis to treat chronic neuroinflammation.

This underlines the need for further investigations to better understand the role of the PD-1 axis during neuroinflammatory disorders.

## Author Contributions

SMan, MO, SMas, AM, and AF wrote the review and correct the text. RF supervised the writing of the review and correct the final form of the article. All authors contributed to the article and approved the submitted version.

## Conflict of Interest

The authors declare that the research was conducted in the absence of any commercial or financial relationships that could be construed as a potential conflict of interest.

## Publisher's Note

All claims expressed in this article are solely those of the authors and do not necessarily represent those of their affiliated organizations, or those of the publisher, the editors and the reviewers. Any product that may be evaluated in this article, or claim that may be made by its manufacturer, is not guaranteed or endorsed by the publisher.
